# Supplementary material for: Physical activity and exercise recommendations for people receiving dialysis: A scoping review
Source: PLoS One. 2022 Apr 28;17(4):e0267290. doi: 10.1371/journal.pone.0267290 (PMC9049336; doi:10.1371/journal.pone.0267290)
Supplement: S1 Table — (DOCX) [file pone.0267290.s001.docx]

Supplementary Information Table 1. Grey literature search strategy

The number of relevant documents retrieved from each source is included in brackets.

| **Journal Home Pages** | **Professional societies** | **Key organisations** |
| --- | --- | --- |
| ***Exercise and Sports Science*** | | |
| British Journal of Sports Medicine (0) | European College of Sport Science (0) | NICE UK (0) |
| Sports Medicine (1) | Exercise and Sports Science Australia (1)* | American College of Sports Medicine (1) |
| American Journal of Sports Medicine (0) | British Association of Sport and Exercise Sciences (1)* |  |
| International Journal of Behavioural Nutrition and Physical Activity (0) | Sport and Exercise Science New Zealand (0) |  |
| Medicine and Science in Sports and Exercise (0) | Canadian Society for Exercise Physiology (0) |  |
| International Journal of Sports Physiology and Performance (0) | South African Sports Medicine Association (0) |  |
| Exercise and Sports Sciences Review (0) | Canadian Kinesiology Alliance (0) |  |
| Journal of Physiotherapy (1) | Australian Physiotherapy Association (0) |  |
| Journal of Science and Medicine in Sport (1) | European Federation of Sports Medicine Association (1) |  |
| Scandinavian Journal of Medicine and Science in Sports (0) |  |  |
| Journal of Sports Management (0) |  |  |
| Applied Ergonomics (0) |  |  |
| Journal of Teaching in Physical Education (0) |  |  |
| Journal of Strength and Conditioning Research (0) |  |  |
| Journal of Head Trauma Rehabilitation (0) |  |  |
| Journal of Sports Sciences (1) |  |  |
| Sports Health (0) |  |  |
| Physical Therapy (0) |  |  |
| Journal of Neurologic Physical Therapy (0) |  |  |
| European Journal of Sports Science (0) |  |  |
| ***Nephrology*** | | |
| Nature Reviews Nephrology (0) | European Renal Association (0) | British Renal Association (1) |
| Journal of the American Society of Nephrology (0) | International Society of Nephrology (0) | National Kidney Foundation (USA) (1)* |
| Kidney International (0) | Chinese Society of Nephrology (0) | Kidney Foundation of Canada (0) |
| Clinical Journal of the American Society of Nephrology (0) | Japanese Society of Nephrology (0) | Kidney Care UK (0) |
| American Journal of Kidney Diseases (0) | Asia Pacific Society of Nephrology (0) | National Kidney Federation UK (0) |
| Nephrology Dialysis Transplantation (0) | Australian New Zealand Society of Nephrology (0) | Kidney Health Australia (2) |
| Kidney International Supplements (0) | Canadian Society of Nephrology (0) | European Renal Association / European dialysis and Transplant Association (0) |
| Seminars in Nephrology (0) | Renal Society of Australasia (0) | Inigo Alvarez de Toledo Foundation/ Fundación Inigo Alvarez de Toledo (1) |
| American Journal of Nephrology (0) | American Society of Nephrology (0) | British Renal Society (0) |
| Minerva Urologica e Nefrologica (0) | South African Renal Society (0) | European Society of Cardiology (1) |
| CKJ: Clinical Kidney Journal (0) | Brazilian Society of Nephrology (0) | Kidney Foundation of Poland/ Krajowa Fundacja Nefrologiczna (0) |
| Advances in Chronic Kidney Disease (0) | Swedish Renal Medicine Association (0) | Foundation for the Development of Nephrology and Transplantation / Fundacja Rozwoju Nefrologii i Transplantacji (0) |
| Kidney International Reports (0) | Spanish Society of Nephrology / Sociedad Española de Nefrología(1*) |  |
| Journal of Nephrology (1) | Argentinian Society of Nephrology / Sociedad Argentina de Nefrología (0) | Amicus Renis Foundation /Fundacja Amicus Renis (2) (2) |
| Current opinion in Nephrology and Hypertension (0) | Colombian Association of Nephrology and Hypertension / La Asociación Colombiana de Nefrología e Hipertensión Arterial (0) | Mexican Institute of Nephrology Research / Instituto Mexicano de Investigaciones en Nefrología (0) |
| Peritoneal Dialysis International (0) | Chilean Society of Nephrology // Sociedad Chilena de Nefrología (2) | Mexican Kidney Foundation / Fundación Mexicana del Riñón (0) |
| Journal of the Spanish Society of Nephrology (1) | Ecuadorian Society of Nephrology / Sociedad Ecuatoriana de Nefrología (0) | The Kidney Foundation of Thailand (0) |
| Indonesian Journal of Kidney and Hypertension (0) | Bolivian Society of Nephrology / Sociedad Boliviana de Nefrología (0) |  |
| African Journal of Nephrology (0) | Peruvian Society of Nephrology / Sociedad Peruana de Nefrología (0) |  |
| Nefrologia al Dia (0) | Uruguayan Society of Nephrology / Sociedad Uruguaya de Nefrología (0) |  |
| Revista Colombiana de Nefrologia (0) | Cuban Society of Nephrology / Sociedad Cubana de Nefrología (0) | Life options rehabilitation advisory council (1) |
| Revista Nefrologia Argentina (0) | Paraguayan Society of Nephrology / Sociedad Paraguay de Nefrología (0) |  |
| Nefrologia Latinoamericana (0) | El Salvador Association of Nephrology and Hypertension Asociación de Nefrología e Hipertensión Arterial de El Salvador (0) |  |
| Revista de Nefrologia, Dialisis y Transplante (0) | Central American and Caribbean Association of Nephrology and Hypertension / Asociación Centroamericana y del Caribe de Nefrología e Hipertensión (0) |  |
|  | Spanish Association of Pediatric Nephrology / Asociación Española de Nefrología Pediátrica (0) |  |
|  | Regional Association of Dialysis and Kidney Transplants of the Federal Capital and Province of Buenos Aires / Asociación Regional de Diálisis y Trasplantes Renales de Capital Federal y Provincia de Buenos Aires (0) |  |
|  | Confederation of Dialysis Associations of the Republic of Argentina / Confederación de Asociaciones de Diálisis de la República Argentina (0) |  |
|  | Dominican Society of Nephrology / Sociedad Dominicana de Nefrología (0) |  |
|  | Spanish Society of Dialysis and Transplantation / Sociedad Española de Diálisis y Trasplantes (0) |  |
|  | Venezuelan Society of Nephrology / Sociedad Venezolana de Nefrología (0) |  |
|  | Latinamerican Society of Nephrology and Hypertension / Sociedad Latinoamericana de Nefrología e Hipertensión (0) |  |
|  | Danish Society of Nephrology (0) |  |
|  | Polish Society of Nephrology/ Polskie Towarzystwo Nefrologiczne (0) |  |
|  | Polish Society of Pediatric Nephrology/ Polskie Towarzystwo Nefrologii Dziecięcej (0) |  |
|  | Malaysian Society of Nephrology (0) |  |
|  | Egyptian Society of Nephrology and Transplantation (0) |  |
|  | African Paediatric Nephrology Association (0) |  |
|  | Mexican College of Nephrologists / Colegio de Nefrólogos de México (0) |  |
|  | Mexican Association of Nurses in Nephrology Asociación Mexicana de Enfermeras en Nefrología (0) |  |
|  | Jordan Society of Nephrology and Renal Transplantation (0) |  |
|  | Emirates Medical Association Nephrology Society (0) |  |
|  | Lebanese Society of Nephrology and Hypertension (0) |  |
|  | Saudi Society of Nephrology (0) |  |
|  | Iranian Society of Nephrology (0) |  |
|  | Kuwait Nephrology Association (0) |  |
|  | Arab Society of Nephrology and Renal Transplantation (0) |  |
|  | National Kidney and Transplant Institute (The Philippines) (0) |  |
|  | The Nephrology Society of Thailand (0) |  |
|  | Myanmar Nephro Uro Society (0) |  |
|  | Korean Society of Nephrology (0) |  |
|  | Nepal Society of Nephrology (0) |  |
|  | Indonesian Society of Nephrology (0) |  |
|  | Indian Society of Nephrology (0) |  |

* Indicates duplicate from database searches
