# Supplementary material for: Physical activity and exercise recommendations for people receiving dialysis: A scoping review
Source: PLoS One. 2022 Apr 28;17(4):e0267290. doi: 10.1371/journal.pone.0267290 (PMC9049336; doi:10.1371/journal.pone.0267290)
Supplement: S2 Table — (DOCX) [file pone.0267290.s002.docx]

Supplementary Table 2. Recommendations for physical activity for people receiving dialysis.

| **Author** | **Recommendation regarding physical activity** | **Suggested examples** | **Timing and Duration** | **Intensity** | **Frequency / progression** |
| --- | --- | --- | --- | --- | --- |
| American College of Sports Medicine ^[30]^ | No detail provided | No detail provided | The duration should be increased by 3–5 min increments weekly until the individual can complete 30 min of continuous activity before increasing the intensity.  Moderate: 30 min+ or  vigorous: 20 min+ per day | Moderate or vigorous but no definition provided | The amount of activity and rate of progression should be guided by an individual patient assessment performed by a qualified staff member (*e.g.* Certified Clinical Exercise Physiologist)  Moderate: 5 days/week or vigorous: 3 days/week |
| Chilean Society of Nephrology ^[28, 29]^ | Perform physical activity | No detail provided | No detail provided | No detail provided | No detail provided |
| European Federation of Sports Medicine Association ^[27]^ | No detail provided | No detail provided | No detail provided | No detail provided | No detail provided |
| Exercise and Sports Science Australia ^[31]^ | No detail provided | No detail provided | No detail provided | No detail provided | No detail provided |
| Fuhrmann and Krause ^[38]^ | No detail provided | No detail provided | No detail provided | No detail provided | No detail provided |
| Heiwe and Jacobson ^[40]^ | No detail provided | No detail provided | No detail provided | No detail provided | No detail provided |
| Isnard- Rouchon et al ^[37]^ | No detail provided | No detail provided | No detail provided | No detail provided | No detail provided |
| Italian Society of Nephrology ^[32]^ | No detail provided | No detail provided | No detail provided | No detail provided | No detail provided |
| KDOQI ^[34]^ | The goal for physical activity should be “cardiovascular exercise” | No detail provided | No detail provided | No detail provided | No detail provided |
| Life Options Rehabilitation Advisory Council ^[26]^ | Encourage enjoyable physical activity to increase participation. | No detail provided | No detail provided | No detail provided | No detail provided |
| Patel et al ^[35]^ | No detail provided | No detail provided | No detail provided | No detail provided | No detail provided |
| Polish Society of Nephrology ^[24, 25]^ | Recreational activity that will contribute to the improvement of cardiovascular and respiratory efficiency. | Walking, Nordic walking, cycling | No detail provided | No detail provided | No detail provided |
| Raj et al ^[36]^ | No detail provided | No detail provided | No detail provided | No detail provided | No detail provided |
| Renal Foundation of Inigo Alvarez de Toledo ^[23]^ | No detail provided | No detail provided | No detail provided | No detail provided | No detail provided |
| Roshanravan et al ^[39]^ | No detail provided | No detail provided | Physical activity recommendations (walking): for sedentary:  20-30 minutes/day   For those highly deconditioned/don’t exercise: 30–60 min/day  For those with sporadic physical activity/ suboptimal exercise/ mildly deconditioned: 30–90min/day | Walking goal for sedentary: 3000-3500/day at light-moderate intensity  For those highly deconditioned/don’t exercise: 3000-4000 steps/day at light- moderate intensity  For those with sporadic physical activity/ suboptimal exercise/ mildly deconditioned: 5400-7900 steps/day at moderate intensity | Physical activity recommendations (walking): 3-5 times/week |
| Spanish Society of Nephrology ^[22]^ | No detail provided | No detail provided | No detail provided | No detail provided | No detail provided |
| UK Renal Association ^[33]^ | No detail provided | No detail provided | No detail provided | No detail provided | No detail provided |
