# Supplementary material for: Physical activity and exercise recommendations for people receiving dialysis: A scoping review
Source: PLoS One. 2022 Apr 28;17(4):e0267290. doi: 10.1371/journal.pone.0267290 (PMC9049336; doi:10.1371/journal.pone.0267290)
Supplement: S3 Table — (DOCX) [file pone.0267290.s003.docx]

Supplementary Table 2. Recommendations for exercise for people receiving dialysis

| Author | Recommendation | Type and mode of exercise | Timing and Duration | Intensity | Frequency / progression |
| --- | --- | --- | --- | --- | --- |
| American College of Sports Medicine ^[30]^ | Programs should consist of a combination of aerobic and resistance training | Aerobic: prolonged rhythmic activities using large muscles eg walking, cycling, swimming  Intradialytic exercise: Pedalling or step device  Resistance: Machines, free weights, bands  Balance and flexibility: static or proprioceptive neuromuscular facilitation  Physical Activity | Aerobic: 20-60 mins continuous activity. If not tolerated 3-5 minute bouts to accumulate 20-60 min per day are recommended  Resistance: 1 set of 10-15 repetition using 8-10 different exercises targeting major muscles  Flexibility: 60 seconds per joint for static (10-30 second hold per stretch); for assisted PNF: 3-6 second contraction followed by 10-30 second | Aerobic: Moderate intensity (40-59% VO2 reserve, RPE 12-13 on a scale of 6-20)  Initial intensity should be light (30-39%VO2R)  HD: Aim to reach RPE 9-13  Resistance: 60-75% of 1 RM. Estimate 1 RM from test of ≥ 3 RM.  Flexibility: Stretch to point of tightness or slight discomfort; PNF: 20-75% of maximum voluntary contraction.  Physical Activity: Moderate intensity | Aerobic: 3-5X/week  Resistance: 2-3X/week  Flexibility: 2-3X/week  PA: 3-5X/week (11th edition)  Progression over time based on individual tolerance.  The amount of activity and rate of progression should be guided by an individual patient assessment performed by a qualified staff member (*e.g.*, ACSM Certified Clinical Exercise Physiologist) |
| Chilean Society of Nephrology ^[28, 29]^ | Perform physical activity | PD Aerobic: Walk, dance, or jog.  HD (patients with hypertension) Aerobic: Walk  HD (patients with dyslipidemia): not specified | PD: 30-40min/ session  HD (patients with hypertension): at least 30 mins  HD (patients with dyslipidemia): not specified | PD: Not specified  HD (patients with hypertension): not specified.  HD (patients with dyslipidemia): moderate | PD: At least 3-4x per week  HD (patients with hypertension): most days  HD (patients with dyslipidemia): not specified |
| European Federation of Sports Medicine Association ^[27]^ | Endurance, interval, muscular endurance, flexibility, balance training and strength training  Dialysis patients may use a bed ergometer (pedalmometer). This is dependent on fitness and needle position. | Aerobic: Jogging, Nordic walking, swimming, skating, aerobics, dance, cycling  Resistance: 80% of 1 RM, 1-2 sets, 8-10 reps of functional training | Moderate intensity: 30-60 minutes per day or 150 minutes per week  Vigorous intensity: 30 minutes per day or 75 minutes per week | Moderate intensity: 40-60% V02 max or RPE 11-13  Vigorous intensity: 60-80% V02 max or RPE 12-18 | 3 times per week |
| Exercise and Sports Science Australia ^[31]^ | Both resistance and aerobic activity should be completed (although not necessarily in the same session). | Aerobic: walking, cycling, jogging, other.  IDE: use seated cycle or arm or leg ergometer  Resistance: weight bearing activity, TheraBands, weight cuffs, light dumbells, weight machines.  Interdialytic Resistance: 8-12 exercises prioritizing major muscle groups  Intradialytic Resistance: up to 12 exercises or as many as possible either “before or during” dialysis  Non Dialysis Days: 8-12 exercises prioritizing major muscle groups  Balance and flexibility: include balance exercise for those at high risk of falls. | Intradialytic exercise to be completed within first 2 hours.  Intra/Interdialytic Aerobic: 30-45 min/session with up to 180min/week  Intradialytic Resistance: 1 set to fatigue or 12-15 reps. To complete either “before or during dialysis.”  Interdialytic Resistance: 1 set to fatigue or 12-15 reps.  “Non Dialysis Day” Resistance (according to patient need): 1 set to fatigue or 10-15 reps.  Balance: no recommendation  Flexibility: 10 minutes per session | Intra/Interdialytic Aerobic: 55–70% max HR, RPE 11–13 moderate (preferably >60% max HR)  Non Dialysis Aerobic (according to patient need): 55–90% max HR, RPE 11–16 moderate to vigorous (60–90% max HR)  Intra/Interdialytic Resistance: 60-70% repetition max    Balance and flexibility: No specific recommendation | Aerobic: No specific recommendation  Resistance: HD: Two non consecutive days  Balance: no recommendation  Flexibility: 5-7x/week |
| Fuhrmann and Krause ^[38]^ | Exercise prescriptions for dialysis patients should be individualized and adjusted to individual capacity | Aerobic: walking, jogging, bicycling, cycle ergometer (home trainer), cross country skiing.  Initial stage: Flexibility, coordination, muscular endurance, and breathing/relaxation exercises.  Build up stage: Flexibility, coordination, muscular endurance, muscular strength, “general endurance”, breathing/relaxation exercises.  Main training stage: flexibility, coordination, muscular endurance, static strength, “general endurance,” “lead up games,” and breathing/ relaxation exercises. | Prior to dialysis or on non dialysis days.  Initial stage: 10-15 min  Build up stage: 20-30min  Main training stage: 40-60 min | 1.Based on target heart rate range maximum:  Initial stage (new patients): 30-50%  Build up stage: 50-60%  Main training stage: 60-75%  2. or use the Borg RPE scale(6-20):  Warm up and cool down: 8-9RPE (light)  Initial phase exercise intensity: 12-13 RPE (somewhat hard) and may not exceed 14-15RPE (hard) | 2+/week. Daily training is optimal.  Gradual progression  Note: “General endurance” during build up stage is defined as interval walking around chairs until breaks are reduced.  “General endurance” during main training phase defined as walk run intervals until walk breaks are reduced. |
| Heiwe and Jacobson ^[40]^ | Not specified | No specific recommendation | More than 30 minutes per session | No specific recommendation | Regular exercise three times per week is beneficial for patients receiving dialysis |
| Isnard- Rouchon et al ^[37]^ | We recommend that all PD programs consider a physical activity program for their PD patients. | Warm up: walk / stationary bike for low or intermediate functioning patients; jumping jacks/walking for high functioning patients  Cardiovascular exercise for low/intermediate functioning patients: Walking / stationary bike  Cardiovascular exercise for high functioning patients: walk/jog or jumping jacks/jump rope.  Upper body for low functioning patients: bicep curls, tricep extension, front raises, and arm scissors.  Upper body for intermediate functioning patients: bicep curls, wall push ups, chest press, and seated rows.  Upper body for high functioning patients: bicep curl, tricep pulldown, shoulder press, and standing rows.  Lower body for low functioning patients: chair squats, calf raises,  Core: trunk twists, seated elbow to knee (lateral bends, standing elbow to knee high functioning)  Cool down: walk / stationary bike / stretch  We encourage PD patients to perform light resistance with weights and encourage swimming in both lakes  and swimming pools | Warm up: 5 minutes  Cardiovascular exercise: 10-20 minutes low functioning / 20-40 minutes intermediate functioning / 40-60 minutes for high functioning patients  Upper body: 1-2 sets of 6-10 reps low functioning / 2-3 sets of 8-12 reps intermediate functioning / 2-3 sets of 8-15 reps high functioning  Lower body: 1-2 sets of 6-10 reps low functioning / 2-3 sets of 8-15 reps intermediate functioning / 2-3 sets of 10-20 reps high functioning  1-2 sets of 6-10 reps low functioning / 2-3 sets of 8-12 reps intermediate functioning / 2-3 sets of 12-15 reps high functioning  Cool down: 5 mins of walking/cycling and 10-15 min stretching | No recommendation for cardiovascular exercise.  Low intensity for resistance training. | No specific recommendation |
| Italian Society of Nephrology ^[32]^ | HD: In stable HD patients, different programs such as home  exercise rehabilitation, supervised exercise training, in hospital gym and intradialytic exercise activity are suggested.  PD: No specific recommendation | Intradialytic cycling  Patients can perform low intensity intradialytic exercises (eg using roller and rubber balls) for coordination, muscle strengthening and flexibility. | Intradialytic cycling: Usually for 30 minutes during the first 2 hours of the HD session.  Duration of each session could be up to 90 min including a warm-up phase of stretching exercises (15–20 min) followed by 20–50 min of cycling on an electronically braked cycle ergometer and finally followed by a recovery phase. | Submaximal (i.e. not exceeding 60 % of peak VO2). | HD: Twice a week on non dialysis days |
| KDOQI ^[34]^ | All dialysis patients should be encouraged to increase physical activity. | ‘Cardiovascular exercise’ | 30 minutes or more | Moderate intensity – at levels appropriate to the capacity, needs, and interest of individual. | Most if not all days of the week.  Sedentary patients should start at very low levels and durations, and gradually progress to recommended levels |
| Life Options Rehabilitation Advisory Council ^[26]^ | Encourage enjoyable physical activity to increase participation.  Exercise program should include activities that will increase cardiovascular fitness, muscle strength and flexibility  Flexibility: gentle stretching | Aerobic: walking, cycling, swimming  Resistance training: resistance band, hand weights, ankle wights or multi-station resistance machines | Aerobic: 30 minutes  Resistance training: Begin with weight that can be comfortably lifted for 10-12 reps. Work up to 10-12 reps x 3 sets | Dependent on individual level of conditioning. Moderate intensity (~60% V02 max)  Individuals with greater fitness can tolerate 80% of maximal capacity  Exercise at RPE of 12-16 on Borg scale (no greater than 16 for individuals who can tolerate moderate activity and no more than 12 in a low intensity program | Aerobic: 3-4 times per week  Progression: build up gradually using several 5 minute sessions each day. Increase by 1-2 minutes each day until able to compete a single 20 minute session. Include rest breaks if needed |
| Patel et al ^[35]^ | Exercise programs should include aerobic and resistance training individualized to goals, circumstances and needs. This includes children and adolescents receiving dialysis. | Suggest utilising guidelines from ACSM^18^  Aerobic: walking and cycling  Resistance:  8–10 different exercises to work the major muscle groups using machines, resistance bands, or free weights | Aerobic: 20-40 min of continuous aerobic/day or 10-minute bouts accumulating 20-60 min/day.  Resistance: 1 set of 10-15 reps. Multiple sets of may be done based on patient tolerance. | Aerobic: Moderate intensity (i.e., 40% to < 60% VO2R or RPE 11–13 on a scale of 6–20). Start at low intensity short duration.  Resistance: 60%-75% of 1 RM. Resistance intensity should start at low loads with higher reps. | Aerobic: 3-5 days/week.  Resistance: 2-3 days/week |
| Polish Society of Nephrology ^[24, 25]^ | It is suggested to perform endurance training in addition to general functional exercise in the form of recreational activity. | Home Exercise Program Aerobic: Walking and cycling  Home Exercise Program Resistance: “general functional exercises.”  PD Aerobic: cycling and walking  PD Resistance: lifting weights. | Home Exercise Program : 30-40 min;  Resistance: repeat each exercise 5-10x (may take 15-30min)  PD: 45-60min (Must pass talk test). | Home Exercise Program: light to moderate (Borg Scale 11-14 or increase in heart rate by 30-40% from resting state).  PD Aerobic: Reach HR intensity as prescribed.  PD Aerobic: Reach HR intensity as prescribed.  PD Resistance: high rep, low weight. | Home Exercise Program: “As often as possible”  PD: 3+ per week. Gradually increase |
| Raj et al ^[36]^ | Exercise programs should include aerobic and resistance training individualized to goals, circumstances and needs for maximal benefit. This includes children and adolescents receiving dialysis. | Suggest utilising guidelines from ACSM for people with CKD  Aerobic: cycling, jogging, running or walking  Resistance training: 8–10 different major muscle free weights, resistance bands or machine | Aerobic: Each exercise session should be 20-60 minutes duration but can accumulate shorter periods for a total of 20-60 minutes.  Resistance: 1 set of 10-15 reps is preferred depending up on persons exercise tolerance. | Moderate intensity (ie, 40% to 59% VO2R or RPE 11–13 on a scale of 6–20)  Resistance: 60-75% of 1 RPM. | Exercise while on dialysis or on non-dialysis days.  Aerobic: 3-5 days/week days  Resistance training: 2 -3 days/week with interval of 48 hours between sessions when training the same muscle group. |
| Renal Foundation of Inigo Alvarez de Toledo ^[23]^ | It is recommended that patients appropriate for intradialytic exercise engage in the treatment. Perform IDE for all 3 treatments if appropriate | Warm up: range of motion of ankles, knees, hips followed by breathing exercises.  Intradialytic aerobic: using pedaler  Intradialytic strength: resistance bands, foam roller, foam ball, ankle weights, pilates circle, and other.  Strength exercises include: hip flexion, hip and knee extension, hip abduction, hip adduction, ankle inversion and eversion.  Cool down: breathing exercises to finish. | Warm up: 4-6min.  Intradialytic aerobic: 2X/week (for those capable of cycling may perform this). For those unable to cycle, they should continue with strength training.  Week 1-2: 10-12min  Week 3-4: 12-15min  Intradialytic strength: 3X/week  Week 1: 1 set of 10 with 60 sec break.  Week 2: 2 sets of 10 with 60 sec break between sets.  Week 3: 2 sets of 10-12 with 60 sec break between sets  Week 4: 3 sets of 10 with 45-60 sec breaks between sets.  All strength: 2 seconds for concentric contraction and 2 seconds for eccentric contraction.  IDE should be started as soon as dialysis treatment is deemed stable by nurse. | Intradialytic aerobic: Borg RPE 10-12  Heart rate monitoring using Karvonen formula  Take Borg at the end of training session. | Progress in a periodized manner in short blocks of 2-5weeks. These are individualized to the patient’s ability. First two weeks no aerobic, rather focus on building strength. |
| Roshanravan et al ^[39]^ | Include aerobic, resistance and flexibility exercises.  For patients who are at risk of falls, balance exercises are advised.  Assess physical performance mid-week on a non-dialysis day | Aerobic: cycling, walking, swimming  Physical activity (walking)  Resistance: multi-joint exercises affecting more than one muscle group and targeting agonist and antagonist muscles  Flexibility exercises: combine with aerobic and resistance when possible. | Aerobic: 20 min/day (or bouts of 3–5 min of intermittent exercise)  AND  Physical activity recommendations (walking): for sedentary:  20-30 minutes/day  For those highly deconditioned/don’t exercise:  30-60min/day  For those with sporadic physical activity/ suboptimal exercise/ mildly deconditioned:  30-90min/day (~150 min/week)  Resistance: minimum of 1 set of 10–15 repetitions; gradually increase to 2–4 sets; choose 8–10 different exercises to work major muscle groups; rest 2–3min between sets; rest>=48hrs between sessions  Flexibility: 10 min/day | Aerobic: Week 1–2: Moderate (RPE 11–13 on scale of 6–20), 55– 70% max HR; Week 3–5: Moderate (RPE 11–16) 55– 90 % of max HR  Walking goal for sedentary: 3000-3500/day at light-moderate intensity    For those highly deconditioned/don’t exercise: 3000-4000 steps/day at light- moderate intensity  For those with sporadic physical activity/ suboptimal exercise/ mildly deconditioned: 5400-7900 steps/day at moderate intensity  Resistance:  Week 1–2: 60–70% of 1-RM;  Week 3–5: 1 RM  Flexibility: not applicable | Exercise training prescriptions should be individualized based on guidelines by ESSA [28] and ACSM 8^th^ edition  Aerobic:  Week 1–2: 2 times per week  Week 3–5: 3 times per week  Goal: 3-5 times per week  Physical activity recommendations(walking): 3-5 times/week  Resistance:  Week 1–5: 2 times per week  Flexibility: 5 x per week |
| Spanish Society of Nephrology ^[22]^ | Encourage exercise along with treatment. | HD Intradialytic Aerobic: cycling  HD Intradialytic Resistance: weights for non vascular arm  HD Interdialytic Aerobic: Walk  HD Interdialytic Flexibility: Stretch arms for those who are in a wheelchair  PD: Not specified | HD intradialytic: not specified.  HD interdialytic: 10-45min  PD: Not specified | HD intradialytic: not specified.  HD interdialytic: not specified.  PD: Not specified | HD intradialytic: not specified.  HD interdialytic: on non dialysis days  PD: Not specified |
| UK Renal Association ^[33]^ | Intradialytic exercise be available in all renal centers for HD patients. | Warm up and cool down recommended  Include aerobic and resistance components of lower or upper body.  Aerobic: Intradialytic cycling  Resistance: TheraBand or ankle weights. | Should be completed between 30 and 120 minutes after commencing HD.  Warm up and cool down for a minimum of 5 min.  Sessions should be 30+ minutes duration | Moderate to vigorous intensity (40-75% VO2 reserve/heart rates reserve or 12-15 on the Borg RPE scale).  Recommended warm up and cool down starting at / or reducing from half the level of intended training intensity | Should be completed during all HD sessions, unless contraindicated.  Volume of exercise to progress gradually.  Once established with intradialytic exercise, additional exercise on non-dialysis days is encouraged. |

Table legend: HD: haemodialysis; UK: United Kingdom; UK: United Kingdom ; KDIGO: Kidney Disease Improving Global Outcomes
