# Supplementary material for: Physical activity and exercise recommendations for people receiving dialysis: A scoping review
Source: PLoS One. 2022 Apr 28;17(4):e0267290. doi: 10.1371/journal.pone.0267290 (PMC9049336; doi:10.1371/journal.pone.0267290)
Supplement: S4 Table — (DOCX) [file pone.0267290.s004.docx]

Supplementary Table 4. Safety and other recommendations

|  | Clearance required | Contraindications to exercise | Precautions for exercise | Specific comments re fistula arm | PD specific comments | Other comments |
| --- | --- | --- | --- | --- | --- | --- |
| American College of Sports Medicine ^[30]^ | Medical clearance required before commencement. | Absolute Contraindications (symptom limited max testing):  Acute myocardial infarction (within 2 days), ongoing unstable angina, uncontrolled cardiac arrhythmia with hemodynamic compromise, active endocarditis, symptomatic severe aortic stenosis, decompensated heart failure, acute pulmonary embolism, pulmonary infarction, or deep venous thrombosis, acute myocarditis or pericarditis, acute aortic dissection, physical disability that precludes safe and adequate testing  Relative Contraindications (symptom limited max testing):  Known obstructive left main coronary artery stenosis, moderate to severe aortic stenosis with uncertain relationship to symptoms, tachyarrhythmias with uncontrolled ventricular rates, acquired advanced or complete heart block, recent stroke or transient ischemia attack, mental impairment with limited ability to cooperate, resting hypertension with systolic >200 mm Hg or diastolic >110 mm Hg, uncorrected medical conditions such as significant anemia, important electrolyte imbalance, and hyperthyroidism | HD: Use RPE instead of HR as an indicator of exercise intensity. Exercise after dialysis may increase risk of hypotension.  HD: Can exercise vascular access arm as long as weight does not directly place pressure on the site itself.  Exercise should be within the first half of dialysis.  Slow progression in cases of medical setbacks. | HD: During dialysis, patients should not exercise the arm with permanent vascular access.  Do not use fistula arm for blood pressure measurement | PD: Exercise and conduct assessment dry to reduce discomfort while exercising | HD: Exercise should ideally be performed on non-dialysis days.  For those unable to do continuous exercise trial a 3 minute work to 3 minute rest ratio until they adapt to training |
| Chilean Society of Nephrology ^[28, 29]^ | Not specified | Not specified | Not specified | Not specified | Not specified | Not specified |
| European Federation of Sports Medicine Association ^[27]^ | No recommendation | No recommendation | No recommendation | No recommendation | No recommendation | No recommendation |
| Exercise and Sports Science Australia ^[31]^ | Thorough medical review including health history and clinical examination. | Contraindications as per the American College of Cardiology and Electrolyte abnormalities – especially hypo/hyperkalaemia; recent changes to the ECG, especially symptomatic tachyarrhythmias or brady-arrhythmias; excess inter-dialytic weight gain >4 kg since last dialysis or exercise session; unstable on dialysis treatment; pulmonary congestion; peripheral oedema. | Regular BP and ECG monitoring recommended during training especially in known hyper or hypokalemia.    Those with diabetic nephropathy and cardio-renal syndrome require closest supervision. | Avoid upper limb activity while AVF healing or temporary vascular access in situ.  DO not use fistula arm for blood pressure measurement or functional assessment | Exercise dry to improve comfort and reduce diaphragmatic pressure, breathlessness and chest discomfort. | Baseline 12 lead ECG during cardiopulmonary exercise testing is recommended. |
| Fuhrmann and Krause ^[38]^ | Sport medical examination and/or cardiorespiratory exercise testing must be performed. | Four contraindications to exercise:  Uncontrolled hypertension; poorly controlled arrhythmia; angina or coronary heart disease at a low workload or at rest; Cardiac insufficiency rated at NYHAII or greater; valve disease > NYHA II; severe retinopathy  Temporary cessation of exercise if: patient has Scribner- Shunt or Shaldon Catheter (temporary catheter)  Repeated hyperkalemia > 6 mmol/L  Severe osteodystrophy (now termed CKD-MBD) (with risk of bone fracture)  Severe peripheral and / or cardiac neuropathy (with risk of injuries) | Do not use hard or dangerous equipment  No jumps from high positions or sports which may lead to injuries.  Avoid Valsalva manoeuvre during static strength exercises  Avoid orthostatic hypotension  Avoid long weight supporting movements  Avoid exercising in hot environments | Protect puncture site with bandage on day of dialysis. Do not wear watches, jewellery on the fistula arm  Do not measure BP on fistula arm  Do not hold fistula arm above head for long periods  Measure pulse at shunt | Observe guidelines for intensity.  Perform exercises partially empty in order to avoid increased pressure in the abdominal cavity  Do not exert pressure on or pull the PD catheter.  Do not perform exercises that involve extensive twisting of the torso or forward spinal flexion.  Do not pull the legs and or knees toward the abdomen.  Breathing should be deep and regular not forced.  Do not perform static strength exercises for the abdominal muscles (dynamic exercises may be performed).  Do not perform exercises lying on stomach or side without support. | Maintain regular breathing especially during strength exercises  Slow down and reduces exertion if presence of: dizziness/lightheadedness, extreme shortness of breath, pain or pressure in chest, pain in muscles or joints, deep red discolouration of face, paleness around nose or mouth, shakiness, or nausea. |
| Heiwe and Jacobson ^[40]^ | No specific recommendation | No specific recommendation | No specific recommendation | No specific recommendation | No specific recommendation | No specific recommendation |
| Isnard- Rouchon et al ^[37]^ | No specific recommendation | No specific recommendation | No specific recommendation | Not applicable | PD patients should clean their access site and change the dressing following swimming. | The theoretical proposal that PD patients have increased intra‐abdominal pressures during various activities, including coughing, straining, and weightlifting may have little clinical relevance. |
| Italian Society of Nephrology ^[32]^ | Structured exercise programs need a pre-evaluation of performance capacity before prescribing the duration and intensity of exercise. Evaluations should include full nutritional status examinations, echocardiography and cardiopulmonary exercise test by a sports medicine physician or cardiologist. | No specific recommendation | No specific recommendation | No specific recommendation | PD: Careful attention paid to activities that increase intra-abdominal pressure (eg strong isometric resistance. exercises) | Exercise sessions should be performed under supervision by appropriately qualified staff. |
| KDOQI ^[34]^ | Evaluation of physical functioning  and re-evaluation of the physical activity program should be done at least every 6 months | No specific recommendation | No specific recommendation | No specific recommendation | No specific recommendation | May need a referral for physical therapy to increase strength and endurance to reach point of adopting recommended levels of physical activity.  Referral to cardiac rehab if patient qualifies. |
| Life Options Rehabilitation Advisory Council ^[26]^ | Patients with CAD or angina require individual assessment | Fever (over 38.3 degrees); when more than one dialysis session has been missed; if the person is medically unstable; if the exercise causes pain  Patients with excessive interdialytic weight gain should avoid exercise before dialysis due to the additional cardiac stress.  Blood glucose <6.1mmol/L or >13.9 mmol/L  Patients with active retinal hemorrhage or recent treatment for retinopathy eg. laser treatments  Severe valvular disease  Patients with renal bone disease may be at risk for fractures and tendon ruptures should avoid high impact activities  Large pericardial effusions should not exercise until appropriate medical management is initiated | Discontinue if unable to maintain effort; unusual SOB; chest pain; nausea; irregular heartbeat; leg cramps; dizziness; muscle or joint pain.  Resistance training: those with poorly controlled HT should use very light weights with increased repetitions and avoid high intensity strength training  Monitor blood glucose before and after exercise if longer than one hour. Consume additional 15-30g of carbohydrate prior to exercise if over 1 hour duration  Insulin requiring patients with diabetes should monitor blood glucose and exercise with a partner  Patients with fluid overload or new to dialysis should be  adequately dialyzed and have fluid balance stabilized before starting an exercise training program. | No specific recommendation | Drain fluid out and exercise when empty or partially empty to permit optimal diaphragm expansion.  Swimming is possible with extra effort to cover the catheter site and clean after to prevent infection. Avoid freshwater lakes and ponds and swim in sea water or chlorinated pool water. | Prescription of exercise intensity according to heart rate does not work in dialysis patients  Warm up for 3-4 minutes at low level and cool down for 2-3 minutes with very light activities to avoid hypotension.  Avoid high intensity exercise immediately post haemodialysis  Patients with extremity amputations should be referred to physical therapy for design of an appropriate program.  Inject insulin into area not being exercised  Patients with neuropathy should be educated about safe foot care |
| Patel et al ^[35]^ | A medical evaluation (assess cardiovascular risk and review medication) and exercise testing are recommended before starting exercise program. | Exercise is contraindicated if SBP >200 mm Hg or DBP >110 mm Hg; electrolyte abnormalities; recent myocardial infarction, or ECG changes. | Use RPE to gauge exertion not heart rate.  HD: Exercise during first half of HD to avoid hypotensive episodes.  Care with patients with unstable potassium | HD: Protect vascular access or AVF site. Can exercise vascular arm as long as weight does not directly place pressure on the site itself. | PD: Dress PD catheter site if swimming  Avoid breath holding during exercise.  Try to exercise with fluid. If not tolerated exercise dry. | All exercise programs require warm up, cool down and flexibility exercises. |
| Polish Society of Nephrology ^[24, 25]^ | Prior to commencing an exercise program, patients should be evaluated by physicians eg nephrologist, cardiologist or diabetologist; and a specialized physical therapist. Patients should be assessed and receive exercise prescription from a specialised physical therapist. | As per Mysliwiec, M. *Wielka Interna-Nefrologia.* 2^nd^ ed.  Medical Tribune Polska. 2017:383-389.  PD related contraindications to exercise: Peritonitis, infection at catheter site, and non-functioning catheter | Avoid high intensity exercise.  PD Patients should avoid the valsalva maneuver, static exercise, and high intensity isometric contractions lasting more than 10 seconds. No sauna, cryotherapy, or contact sports are recommended. For water sports, secure catheter with an extra stoma bag. | Do not overstress arm with fistula. | Initial training sessions should be short and monitored 1:1 with the therapist. Begin and end each session with a 5-10min warm up and 5-10 min cool down.  Patients should attempt to exercise with full peritoneal cavity (up to 2L). Only patients with hernias, leaks or hypotension during exercise should consider exercising with an empty cavity.  Exercising core is encouraged as this may reduce risk of inguinal and umbilical hernias. It is not recommended to perform intense and long contractions of abdominal muscles. | HR intensity is prescribed based on an ECG stress test. It is best to monitor home exercising patients via telemetry.  Home Exercise Program: Best to do on non-dialysis days. For patients who are physically unable to perform 30-40min, or those who perceive themselves weaker on a given day, should exercise 10-15min.  Terminate exercise sessions with the inability to maintain intensity, significant shortness of breath, severe facial flushing, paleness or cyanosis. Terminate session if patient reports angina, nausea, arrhythmias, joint or muscle pain. |
| Raj et al ^[36]^ | A thorough medical examination and response to stress testing are suggested before initiating any exercise regimen | No specific recommendation | Use RPE to gauge exertion not heart rate.  HD: Exercise during first half of HD to avoid hypotensive episodes. | HD: Can exercise vascular access arm as long as weight does not directly place pressure on the site itself. | PD: Try to exercise with fluid. If not tolerated exercise dry. | All exercise programs require warm up, cool down and flexibility exercises. |
| Renal Foundation of Inigo Alvarez de Toledo ^[23]^ | The nephrologist is responsible for medical clearance prior to prescribing intradialytic exercise. Physician will ensure stable clinical status and assess: blood pressure, hydration, blood glucose, adequate dialysis dose, cardiovascular status, and anemia.  Articular assessment prior to physical assessment  Specialized “physical educator” to complete physical assessments. | Not specified | Terminate if patient experiences:  drowsiness, dizziness, or physical discomfort.  If cramping or joint pain occurs eliminate movement causing the aggravation.  Consult nephrologist and advise nurse of hypertension (SBP >10-15% of usual SBP), hypotensive (<10% of normal DBP), or if resting heart rate is high (>20% of usual HR). With the physician’s consent exercise may continue.  With strength training avoid valsalva maneuver due to increase in blood pressure.  Patients who have a hypotensive tendency should begin IDE as early into dialysis treatment as possible.  All patients should wait for nurse to confirm when HD treatment is considered stable prior to beginning exercise.  Measure pre-exercise blood glucose. Considered ideal between 90-150 mg/dL (5.0-8.3mmol/L)  Movements causing muscular or articular pain should be removed from the program.  Those with osteoarthritis should avoid forced rotations. | Not specified | Not applicable | Patients categorized into level 1 (higher functioning) and level 2 (lower functioning)  Guidelines must be adapted to individual patient.  If musculoskeletal injury occurs, the physician will provide clearance. If physiotherapy is required, IDE will be paused until rehabilitation is complete. IDE to resume progressively.    Those with OA: focus on frequency over intensity  Those with spinal issues: focus on achieving good posture prior to beginning any exercise program |
| Roshanravan et al ^[39]^ | All patients should have a medical evaluation, physical assessment, assessment of CV risk and assessment of current physical function.  Physical performance should be assessed for the HD patient mid-week on a non-dialysis day. | Absolute contraindications to exercise include unstable CHD, decompensated heart failure, uncontrolled arrhythmias, severe pulmonary hypertension (mean pulmonary arterial pressure >55mm Hg), severe and symptomatic aortic stenosis, uncontrolled HT (>180/110), and aortic dissection.  Exercise should be stopped if patients develop excessive shortness of breath, angina, severe headache or dizziness. | Patients receiving vasodilators such as alpha blockers, calcium channel blockers, central alpha agonists (clonidine), nitrates and hydralazine may develop hypotension after exercise. A prolonged cool down is recommended.  Patients on beta blockers: 1) May develop hypoglycemia when exercising in hot and humid environments. Exercise intensity and duration should be reduced. 2) May have reduced submaximal and maximal exercise capacity warranting RPE to monitor intensity.  In patients prone to hypoglycemia, glucose levels should be checked before and after exercise and they should have high glycemic index snacks readily available. | HD: Exercise can be performed in the arm or leg with dialysis access once the access has healed but this limb should not be exercised during dialysis. | PD: Some patients experience abdominal discomfort due to contact between the dialysis catheter and abdominal viscus. To manage this keep enough fluid in the peritoneum to allow for floating of the catheter for those without day dwells  PD: Patients can achieve higher levels of exertion when the abdomen is empty; however, this factor should not discourage exercise among PD patients who require daytime dwells. | Subjective and objective assessment of preclinical mobility disability is recommended because of the high risk of decline in mobility.  Prescriptions should be individualized. |
| Spanish Society of Nephrology ^[22]^ | Not specified | Not specified | Not specified | Not specified | Swimming in waters with high amounts of germs, such as lakes and ponds, in not recommended. Pedalling using an upright bike should be monitored closely to prevent an increase in pressure at catheter site. | None |
| UK Renal Association ^[33]^ | No specific recommendation | New to HD (within 3 months); has uncontrolled infection or fever; recent MI (within 8 weeks) or undiagnosed chest pain; unstable ischemia, heart failure(decompensated); uncontrolled arrhythmias; severe and symptomatic aortic stenosis; hypertrophic cardiomyopathy/cardiomyopathy from recent myocarditis; severe pulmonary hypertension; active or suspected myocarditis/pericarditis; suspected or known dissecting aneurysm; thrombophlebitis; recent systemic or pulmonary embolus; DVT; Excessive IDWG; Blood pressure > 160/100 or heart rate > 100 bpm; SOB or substantial peripheral oedema; BGL > 16.7 mmol/L and ketotic; BGL < 5.5 mmol/L | Prior to exercise: check subjective readiness to exercise, BP, HR and BGL  Monitor during exercise for pain, excessive fatigue, altered consciousness, breathlessness, dizziness, chest pain, anxiety, and cyanosis.  BP must not exceed 220/105mm Hg / RPE on Borg Scale must not exceed 15 (hard / heavy)  Post exercise monitor BP, HR and observe for 20 minutes. Be aware of hypotension during remainder of dialysis session.  If hypoglycaemia occurs in diabetics, administer 15g of carbs and reassess BG L. Repeat until BGL >5.5mmol/L | No specific recommendation | No specific recommendation | Exercise should be supervised for greatest compliance by an appropriately trained individual. |
