# Supplementary material for: Physical activity and exercise recommendations for people receiving dialysis: A scoping review
Source: PLoS One. 2022 Apr 28;17(4):e0267290. doi: 10.1371/journal.pone.0267290 (PMC9049336; doi:10.1371/journal.pone.0267290)
Supplement: Attachment — Submitted filename: response to reviewers v2.docx [file pone.0267290.s006.docx]

Response to reviewers

22/1/2022

Dear Editor,

We are pleased to submit the revised article for consideration for publication in your journal. Thank you to both reviewers and the editor for the useful feedback and very fast turnaround during the end of year period.

We have made a number of changes in response to reviewer comments / suggestions and these are shown in track changes or red colour in the revised version.

In response to the query about funding, as directed we would like to include the proposed amended statement for funding: *Funding was received from the Illawarra Health and Medical Research Institute for this work. The funders had no role in study design, data collection and analysis, decision to publish, or preparation of the manuscript”*

Yours Sincerely,

Dr Kelly Lambert

Journal requirements

**We note that Figure 2 in your submission contain [map/satellite] images which may be copyrighted. All PLOS content is published under the Creative Commons Attribution License (CC BY 4.0), which means that the manuscript, images, and Supporting Information files will be freely available online, and any third party is permitted to access, download, copy, distribute, and use these materials in any way, even commercially, with proper attribution.**

**We require you to either (1) present written permission from the copyright holder to publish these figures specifically under the CC BY 4.0 license, or (2) remove the figures from your submission:
   
Please upload the completed Content Permission Form or other proof of granted permissions as an "Other" file with your submission.**

Response: The mapchart.net website (<https://mapchart.net/feedback.html>) contains a specific statement that “*users are allowed to use, edit and modify any map created with MapChart for private or commercial use/publication freely, by adding any kind of reference to MapChart in your work. Some examples of an appropriate reference:*

- *Keeping the Created with mapchart.net tag that generated maps have embedded.*
- *A simple link to the front page or any other page of MapChart.*
- *A text attribution on the page or inside the article/publication where the map is used (as a caption, in the credits, etc).*

*This work is licensed under a* [*Creative Commons Attribution-ShareAlike 4.0 International License*](http://creativecommons.org/licenses/by-sa/4.0/)*.”*

We have therefore added additional information to Figure 2 which states: “Image created using mapchart.net under a Creative Commons 4.0 International License”

**Additional Editors Comments**

**I agree with reviewers’ comments about tables 2 to 4. These tables are cumbersome, not easy to read, and just a repetition of the text. Table 5 must be kept.**

Tables 2-4 have been changed to Supplementary Tables based on reviewer feedback. We feel some readers may be interested in the granular detail provided so have elected to keep as supplementary material rather than remove entirely or reformat.

Previous Table 5 has been retained and is reformatted to include the evidence level of the recommendations retrieved and is now labelled Table 3

A new table has been included which summarises the recommendations (a ‘super’ summary). This is labelled as Table 2 in the revised version and we hope this provides more clarity and improves the reader experience compared to the original version.

**I question the fact to consider at the same level recommendations from authors (ref 34 and 35 as an example) and recommendations from national societies.**

We acknowledge the challenge of the reviewers when attempting to discern the recommendations described in the results section. To alleviate this, we have included a column in Table 3 which provides a rating of the quality of evidence and the strength of recommendations contained within the publications retrieved. In addition, in the new Table 2, we have bolded recommendations retrieved via a systematic review process so as to highlight recommendations based on opinion only. We hope this is helpful for readers.

**I share the concerns of Reviewer 3 about the “recommendations on recommendations”. The authors should only focus on the clear result of their literature review, i.e. the heterogeneity in the recommendations and the need for further researches in the field.**

We have changed the subheading in the discussion to future directions. This is to clarify that we are not making recommendations on recommendations, but rather, are making suggestions for future work that have been identified from this review

**All points that could be considered as “personal opinion” (even if of interest) should be deleted or labeled as “opinion of the authors”.**

To help address this point, we have highlighted in the new Table 2, recommendations that were retrieved via a systematic review process (as opposed to opinion only). We hope this addresses the reviewer’s concern.

**I also agree on the importance of describing the “level of evidence” in recommendations when available.**

The strength of the recommendations retrieved from the 19 publications is summarised in Table 3 and also now described in the manuscript (lines 415-418, 435-437):

*Table 3 provides a condensed overview of the guidance found in the publications retrieved and a rating of the quality of evidence and the strength of recommendations contained within these publications. Fifteen publications provided opinion level guidance, while four produced guidance produced by a systematic review of the evidence.*

**Reviewer comments**

**Reviewer #1: 
- In the introduction, please detail the benefits that are expected from exercises and physical activities in this population. Please also detail a bit more their adverse effects.**

**Additional wording has now been included. *“****Remaining physically active is important to patients undertaking dialysis [6-9]. Exercise also produces many benefits including improved physical function [3], muscle mass and strength[10]. However, there are numerous barriers to undertaking regular physical activity…”*

**- Some of the included publications were published more than 15 years ago. I would suggest limiting the selection to at least the 10 past years only, if authors want their work to reflect the current state of art.**
Thank you for the observation. We have elected to keep all eligible publications as these may still be used in clinical practice despite their vintage. We have however included reference to the nature of the age of these older publications in the results section lines 158-159. We have also updated new Table 3 with the year of publication.

**- Tables 2-3-4 are very long and somehow redundant with the text. They should rather be inserted as supplemental material. On the contrary, it could be interesting to add a table summarizing the common recommendations among the included publications. Such table could also include the major ranges of differences, as explain in the results. It could help the reader to get an overview about what should be done with the patients, even if there are lots of gaps and discrepancies in the included publications.**

Thank you for the suggestion / feedback. We have elected to move the table 2-4 to supplementary information as we believe some readers may be interested in the detail provided. We have also summarised this information into one table (new Table 2). We hope this provides clarity and a concise overview.  **- L459: please correct the sentence: “ … with the USE (?) of …”**
Thank you – this is now corrected

**- L506: please specify that these recommendations you mention are related to future works**

Thank you – we have now amended

**Reviewer #2:** no comments to address. Thank you for the feedback

**Reviewer #3: The paper is a review of recommendations on physical activity and exercise for people receiving dialysis. Although literature has been widely studied, the paper is a list of existing recommendations and it does not bring much except in concluding that recommendations are very different one from another. A more important question to be answered would be: why do these recommendations vary so much? Which studies are they based on?**

To address this concern, we have undertaken additional synthesis and this is shown in Table 2. We believe this additional summary of what was previously contained in tables 2-4, is a more useful method to gauge the areas where future work can be undertaken to improve the quality of advice on exercise for patients undertaking dialysis.

**To finish, the authors provide interesting advices and a checklist for future recommendations. These “recommendations for the recommendations” are common sense but they do not include any reference and are mainly the author’s opinion. There again, a more important question to be raised could be: which trials are lacking to improve the grade recommendation and to better define which physical activity and exercise is beneficial to the patients.**
We have altered this section of the manuscript to ‘Future recommendations’ . We believe this better reflects the intent of this paragraph and is more aligned with the intention of the scoping review. In addition, we have now highlighted which of the guidance retrieved is based on systematic review evidence. While some sentences in this section may be common sense, we believe this is the first time, all previous guidance has been synthesised in such a manner.

**The level of evidence and grade of recommendation should be provided for each recommendation when available.**

The level of evidence has now been included in the new Table 3. Reference to the level of evidence is also included in the results lines 415-418 and discussion line 435-437.

*Table 3 provides a condensed overview of the guidance found in the publications retrieved and a rating of the quality of evidence and the strength of recommendations contained within these publications. Fifteen publications provided opinion level guidance, while four produced guidance produced by a systematic review of the evidence.*

**Table 2 does not bring much. It is large and not easy to read and could be replaced by text.**

We have elected to move the table 2-4 to supplementary information as we believe some readers may be interested in the detail provided. As described above, we have summarised information from the original tables 2-4 into one table (new Table 2). We hope this provides clarity and a concise overview. **Paragraph on safety recommendations should be re-written and include the safety recommendations. To only describe the number of papers providing such data is not very interesting.**

Thank you – this has been amended in the new Table 2 to be more informative and useful for readers.

**The authors should define in the introduction or materials (rather than in the discussion) the terms “physical activity” and “exercise” and explain the difference.**
These definitions have been moved from the discussion and included in the methods (line 134-137). Thank you for the suggestion.
