# Supplementary material for: Physical activity and exercise recommendations for people receiving dialysis: A scoping review
Source: PLoS One. 2022 Apr 28;17(4):e0267290. doi: 10.1371/journal.pone.0267290 (PMC9049336; doi:10.1371/journal.pone.0267290)
Supplement: Attachment — Submitted filename: response to reviewers v2.docx [file pone.0267290.s007.docx]

Response to reviewers

4/4/2022

Dear Editor,

We are pleased to submit the revised article for consideration for publication in your journal. Thank you to both reviewers for the useful feedback.

We have made a number of changes in response to reviewer comments / suggestions and these are shown in yellow in the revised version.

Yours Sincerely,

Dr Kelly Lambert

**Reviewer 3 comments**
**1. I very much appreciated the paragraphs on the “recommendations regarding precautions for exercise”. To better understand which patient (dialysis, PD and HD) is concerned by the precautions, maybe this paragraph could be separated in 3 subtitles:
- Common risks for dialysis patients (potassium, warming and cooling, monitoring intensity...)
- Specific risks for hemodialysis patients (avoid exercise after HD session, AVF related risks etc...)
- Specific risks for PD patients (intrabdominal pressure, access site precautions...)**

Thank you for this helpful suggestion. The changes suggested have been included and the subheadings are shown in yellow in the revised version.

**2. Also, the risks of exercising in hemodialysis and peritoneal dialysis patients are not explained: for example**
**- Line 368: Could the authors be more explicit on the risk of increasing intra-abdominal pressure: Is it for dialysate reabsorption? For hernias?**

As this is a review, we were very careful in only presenting the recommendations that were documented in the reviewed manuscripts. The risk/s of increased intra-abdominal pressure was not explicitly detailed in the cited sources, potentially given limited evidence in this space. Whilst one could postulate that increasing intra-abdominal pressure could promote hernia formation and diaphragmatic splinting, we do not believe that including such comments is within the scope of this review. In addition we have included a further citation that stated “this effect may be of little clinical relevance”.

**- Line 401: Access Site Precautions for Patients. What could be the risk if weight is directly placed onto the AVF? Does it increase the risk of bleeding from fistula?**

Not placing weights on AVF would be part of normal care and routine patient education - including no tight clothes, watches, avoidance of sharp objects etc. The risks of placing a weight on the AVF include increased risk of thrombosis, bleeding and potentially infection. As above, including these potential mechanisms appears to be outside the scope of this review. Common sense would suggest not placing weights o AVF and we have not added to this.

**3. Table 2: Contraindications: The list of contra-indications is not clear.
- For example, “high BP >160/100mm/Hg” is proposed as a contra-indication, but is it chronic hypertension or the blood pressure at the moment of the exercise?**

This is stating the BP at the time of exercise, similar to other contraindications. The word “Prevalent” has been added to help clarity. It now reads: “***New within 3 months to hemodialysis, hypo or hyperglycaemia, high prevalent BP >160/100mm/Hg,*** ***SOB, peripheral oedema, suspected or recent pericarditis, myocarditis, dissecting aneurysm, DVT, not stable on HD (eg intradialytic hypotension),...”***

**- “Not stable on HD” : what does this mean? Is it intradialytic hypotensive episodes?**

**This refers to intradialytic hypotension. The word has been added to the recommendation.**

**- “uncontrolled HT >180/110” : what does HT mean? Is it hypertension? Then is it 160/110 or 180/110? This point is clear in the text (lines 303-305), but not in the Table.
This section could be simplified, maybe by sorting contra-indications.**
Thank you for pointing this abbreviation out. HT refers to hypertension and has been updated in the table accordingly.

**Minor Comments**
**1. Table 2: Precautions to Exercise: “Avoid exercise in hot environment s including saunas” Please correct the typo.**

This has now been corrected

**2. It is not clear to me whether blood pressure must be measured before exercise sessions and if it might be a contra-indication.**
Hypertension and Intradialytic hypotension have been stated as potential contraindications. Blood pressure is measured frequently pre, during and post-dialysis so this is a part of standard care.

**3. Table 2: Legend:- “Bolded statements are based on higher quality evidence rather than opinion only”: please be more precise on the difference of evidence quality between bolded and non bolded statements. Is it the number of papers with the statement or a grade of level evidence (then precise)?**

We have now clarified this with the statement: Bolded statements are derived from recommendations using systematic reviews or randomised controlled trials (higher quality evidence) rather than opinion only.

**4. Line 277 “Where included, the most common recommendation was avoid placing weights on the fistula site (Table 2).” Please correct typo error**

This has now been corrected

**5. Line 279: “For people undertaking PD guidance was provided to avoid increasing intra-abdoinal pressure and commentary given on fill volume).” Please correct typo error**

This has now been corrected
